# Diversity and Origin of Dengue Virus Serotypes 1, 2, and 3, Bhutan

**DOI:** 10.3201/eid1510.090123

**Published:** 2009-10

**Authors:** Tandin Dorji, In-Kyu Yoon, Edward C. Holmes, Sonam Wangchuk, Tashi Tobgay, Ananda Nisalak, Piyawan Chinnawirotpisan, Kanittha Sangkachantaranon, Robert V. Gibbons, Richard G. Jarman

**Affiliations:** Ministry of Health, Thimphu, Bhutan (T. Dorji, S. Wangchuk); United States Army Medical Command–Armed Forces Research Institute of Medical Sciences, Bangkok, Thailand (I.-K. Yoon, A. Nisalak, P. Chinnawirotpisan, K. Sangkachantaranon, R.V. Gibbons, R.G. Jarman); The Pennsylvania State University, University Park, Pennsylvania, USA (E.C. Holmes); National Institutes of Health, Bethesda, Maryland, USA (E.C. Holmes); Ministry of Health, Gelephu, Bhutan (T. Tobgay).

**Keywords:** Dengue, Bhutan, Nepal, phylogeny, emergence, serotypes, viruses, dispatch

## Abstract

To determine the serotype and genotype of dengue virus (DENV) in Bhutan, we conducted phylogenetic analyses of complete envelope gene sequences. DENV-2 (Cosmopolitan genotype) predominated in 2004, and DENV-3 (genotype III) predominated in 2005–2006; these viruses were imported from India. Primary dengue infections outnumbered secondary infections, suggesting recent emergence.

Dengue infections have increased worldwide in recent decades. Before 1970, only 9 countries had experienced epidemics of dengue hemorrhagic fever; by 1996, this number had increased to 102 (*1*). Dengue is endemic to most of Southeast Asia; high numbers of cases are reported each year in Laos, Cambodia, Vietnam, Singapore, and Thailand. Dengue was first reported in Nepal in 2004 (*1*). Serologic testing of a group of febrile patients in Nepal showed that 8% had immunoglobulin (Ig) M against dengue, and a recent report noted 11 serologically confirmed dengue cases in 2006 (*2*,*3*). The Armed Forces Institute of Medical Sciences (AFRIMS) in Bangkok, Thailand, recently confirmed the presence of all 4 dengue serotypes in Nepal (*4*).

In another Himalayan nation, Bhutan, dengue was first suspected in the summer of 2004. Bhutan is an extremely rugged and mountainous country of 38,394 km^2^ with an altitude ranging from 150 m on the southern border with India to >7,000 m in the mountains bordering Tibet (Appendix Figure). The dengue outbreak resulted in 2,579 cases (*5*), almost all of which occurred in and around Phuntsholing district (2005 census population 20,537) in southern Bhutan on the border with India. Fifty-two serum samples from this outbreak were tested by using dengue enzyme immunoassay (EIA) at the National Institute of Communicable Diseases, New Delhi, India. Twelve (23%) samples were positive for antidengue IgM. Thirty-five (67%) of the 52 samples also were tested at Suraksha Hospital, Kolkata, India, of which 5 were positive.

Since the initial report in 2004, fewer clinical cases were reported from Bhutan in 2005 and 2006; dengue virus 3 (DENV)-3 was the dominant serotype (*5*). Although some serologic analysis is available, serum samples from Bhutan have not been evaluated for dengue by using molecular techniques. In particular, circulating DENV in Bhutan has not been genetically characterized, so from where and how frequently DENV is imported into Bhutan are unclear.

## The Study

Acute-phase blood samples were collected from persons with suspected dengue in and around Phuntsholing during 3 periods in 2004–2006 (June 15–August 2, 2004; September 8–December 2, 2005; and July 19–November 30, 2006). All samples were collected from hospitalized patients in whom dengue infection was diagnosed on the basis of clinical signs and symptoms. Convalescent-phase samples were not available. Because all samples were collected by the Bhutan Ministry of Health as part of a public health effort, the study did not require institutional review board–approved human-use protocol.

A total of 168 samples (53 from 2004, 19 from 2005, and 96 from 2006) were delivered to AFRIMS for confirmatory dengue testing. Samples were tested by using reverse transcription–PCR/nested PCR modified from published methods (*6*) and the in-house dengue IgM/IgG EIA (*7*). In addition, the complete envelope (E) gene was sequenced for samples that were dengue positive by PCR. Two DENV-2–positive samples from 2004 and 19 DENV-3–positive samples from 2005–2006 underwent E gene sequencing. Sequencing also was attempted on additional PCR-positive samples but was not successful because of insufficient genetic material. All dengue E gene sequences from the study have been submitted to GenBank and assigned accession nos. FJ606692–FJ606712.

The E gene sequences generated here were combined with homologous sequences from 262 isolates of DENV-2 (total data set of 264 sequences, 1,485 nt) and 245 isolates of DENV-3 (total data set of 264 sequences, 1,479 nt), representing the full phylogenetic spectrum of these viruses in humans. Maximum-likelihood (ML) phylogenetic trees were estimated for both datasets using the method implemented in the PAUP* package (*8*). The general time reversible + I + Γ_4_ model of nucleotide substitution was the best fit to DENV-2 and DENV-4 as determined by MODELTEST (*9*). A neighbor-joining bootstrap resampling analysis (1,000 replications) also was performed to assess support for specific nodes, again by using the ML substitution model.

A total of 168 samples from persons suspected to have dengue were tested at AFRIMS. DENV infection was confirmed by PCR or EIA for 34 (64%) of 53 samples from 2004, 12 (63%) of 19 samples from 2005, and 57 (59%) of 96 samples from 2006 (combined 103 [61%] of 168 positive). PCR testing showed 7 samples from 2004 were DENV-2; 4 samples from 2005 were DENV-3; and from 2006, 40 were DENV-3 and 1 was DENV-1 (combined 52 [31%] of 168 PCR positive).

Serologic testing indicated that 28 samples from 2004 were positive for dengue by EIA; 15 were primary infections, and 13 were secondary infections according to published criteria (*7*). Ten samples from 2005 were EIA positive; 8 were primary infections, and 2 were secondary infections. Thirty-nine samples from 2006 were EIA positive; 23 were primary infections, and 16 were secondary infections (Table).

**Table:** Laboratory-confirmed dengue cases, Bhutan, 2004–2006*

Year	Dengue cases			PCR serologic results	
	Primary	Secondary		Positive	Negative
2004	15	13 (1 DENV-2)		6 (DENV-2)	19
2005	8 (2 DENV-3)	2		2 (DENV-3)	7
2006	23 (13 DENV-3)	16 (11 DENV-3)		17 (16 DENV-3, 1 DENV-1)	40

*DENV, dengue virus.

Clinical information for these dengue-positive samples was limited. However, mean age of the 103 patients with laboratory-confirmed dengue was 31 years (32 years in 2004, 28 years in 2005, and 30 years in 2006) (range 2–69 years). Mean age of the 46 patients with primary dengue infection was 29 years; that of the 31 patients with secondary dengue infections was 32 years.

**Figure 1:**
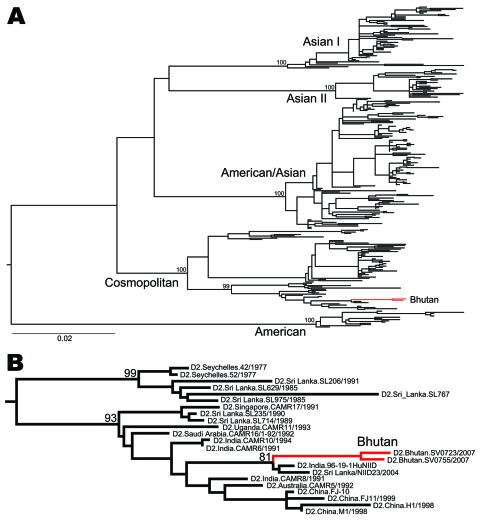
A) Maximum likelihood phylogenetic tree of 264 complete envelope gene sequences of dengue virus serotype 2 (DENV-2). The different genotypes of DENV-2 and the isolates from Bhutan (red) are indicated. Scale bar indicates number of substitutions per site. B) Magnification of the part of the phylogeny where the Bhutan sequences (red) fall. The tree is midpoint rooted for clarity only, and all horizontal branch lengths are drawn to a scale of nucleotide substitutions per site. Bootstrap support values are shown for key nodes only.

**Figure 2:**
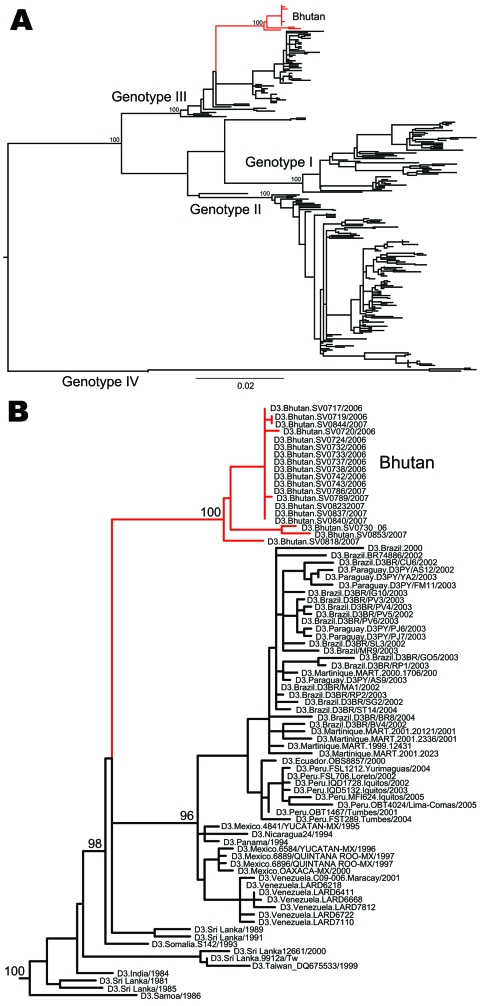
A) Maximum-likelihood phylogenetic tree of 264 complete envelope gene sequences of dengue virus serotype 3 (DENV-3). The different genotypes of DENV-3 and the isolates from Bhutan (red) are indicated. Scale bar indicates number of substitutions per site. B) Magnification of the part of the phylogeny where the Bhutan sequences (red) fall. The tree is midpoint rooted for clarity only, and all horizontal branch lengths are drawn to a scale of nucleotide substitutions per site. Bootstrap support values are shown for key nodes only.

Phylogenetic analysis of complete E gene sequences indicated that the 2 DENV-2 isolates sampled in 2004 fell into the Cosmopolitan genotype, which is widely distributed throughout the tropical and subtropical world (Figure 1). These viruses were closely related to those sampled in a similar period in the Indian subcontinent (India and Sri Lanka), suggesting these DENV-2 isolates were imported into Bhutan from the Indian subcontinent. A remarkably similar picture was seen for DENV-3 (Figure 2). The 19 DENV-3 isolates sampled from 2005 and 2006 clustered closely within viral genotype III, which is found in Africa, the Western Hemisphere, and the Indian subcontinent. Again, the DENV-3 phylogeny is compatible with the migration of this virus northward through India to Bhutan.

## Conclusions

Our genetic characterization of DENV in Bhutan demonstrates that at least 3 DENV serotypes have circulated there. The E gene sequence data for the 2 DENV-2 strains from 2004 and the 19 DENV-3 strains from 2005 and 2006 indicate that the DENV strains in Bhutan are similar to those circulating regionally and thus were most likely introduced (or reintroduced) from neighboring areas. That such emergence in Bhutan has occurred only recently is further supported by the observation of more primary than secondary dengue infections for all 3 years, as well as the relatively advanced mean age of patients.

## Supplementary Material

Appendix Figure Map of Bhutan. Selected cites are indicated by enclosed circle and elevation of the city in meters in parentheses.
