# Supplementary figures and images for: Diversity and Origin of Dengue Virus Serotypes 1, 2, and 3, Bhutan
Source: Emerg Infect Dis. 2009 Oct;15(10):1630–2. doi: 10.3201/eid1510.090123 (PMC2866390; doi:10.3201/eid1510.090123)

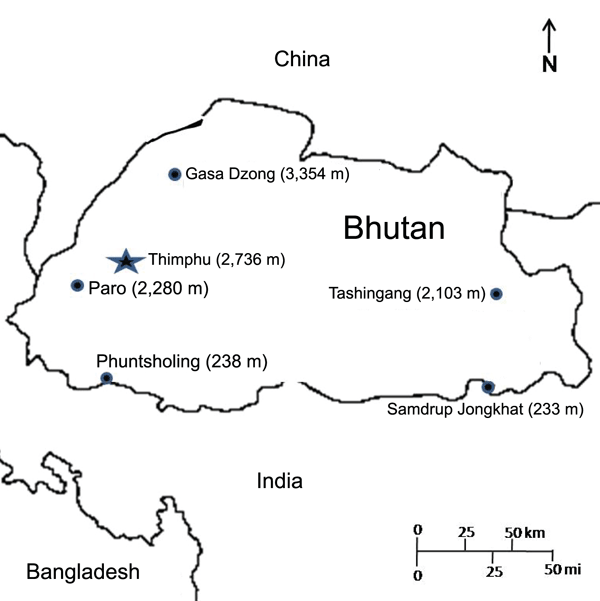

Supplement: Appendix Figure — Map of Bhutan. Selected cites are indicated by enclosed circle and elevation of the city in meters in parentheses. [file 09-0123_appF-s1.gif]
